# Mobile Health Interventions for Enhancing Awareness and Screening of Diabetic Retinopathy: A Narrative Review of Behavior Change Strategies, Digital Health Ecosystems, and Implementation Frameworks

**DOI:** 10.7759/cureus.113162

**Published:** 2026-07-22

**Authors:** Animesh Rai, Anandhi Ramachandran

**Affiliations:** 1 Non-Communicable Diseases and Maternal and Child Nutrition, Vitamin Angels, Lucknow, IND; 2 Health Management, International Institute of Health Management Research, New Delhi, IND

**Keywords:** behavior change communication, diabetic retinopathy, health literacy, mobile health, retinal screening

## Abstract

Diabetes mellitus continues to be one of the fastest-growing non-communicable diseases worldwide and is accompanied by an increasing burden of long-term microvascular complications, among which diabetic retinopathy remains one of the leading causes of preventable blindness among working-age adults. Because early-stage diabetic retinopathy frequently progresses without noticeable visual symptoms, timely retinal screening remains essential for preventing irreversible vision loss. Nevertheless, awareness of diabetic retinopathy, knowledge of recommended retinal examinations, and participation in preventive eye care services remain inadequate, particularly among individuals living in rural and resource-constrained settings.

The rapid expansion of digital technologies has created new opportunities for delivering health communication through mobile health (mHealth) interventions that are accessible, scalable, and capable of reaching geographically dispersed populations. Mobile-based communication strategies have demonstrated considerable potential for improving health knowledge, reinforcing preventive behaviors, supporting treatment adherence, and facilitating greater engagement with healthcare services. However, although the effectiveness of mHealth interventions has been demonstrated for several chronic diseases, evidence specifically addressing diabetic retinopathy awareness and retinal screening remains comparatively limited.

This narrative review critically synthesizes the available evidence on diabetic retinopathy awareness, screening behavior, barriers to preventive eye care, behavior change theories, mHealth interventions, digital health ecosystems, technology adoption models, and implementation frameworks relevant to diabetic retinopathy prevention. Evidence from public health, ophthalmology, behavioral science, digital health, and implementation research was examined to provide a comprehensive understanding of the factors influencing awareness, preventive health behavior, and retinal screening among individuals living with diabetes.

The available evidence indicates that preventive eye care practices are influenced by a complex interaction of cognitive, behavioral, social, cultural, socioeconomic, and healthcare system determinants. Behavioral theories provide important conceptual foundations for designing communication strategies that extend beyond information dissemination to strengthen motivation, improve perceived susceptibility, reduce behavioral barriers, and encourage sustained participation in retinal screening. Simultaneously, implementation science frameworks provide structured approaches for improving the adoption, implementation, scalability, and sustainability of mHealth interventions within existing healthcare systems.

Overall, the available evidence suggests that mobile-based behavior change interventions represent a promising strategy for strengthening diabetic retinopathy awareness, improving retinal screening uptake, and supporting preventive eye care among individuals living with diabetes. Future research should focus on culturally appropriate, theory-informed, and implementation-oriented mHealth interventions that are rigorously evaluated for effectiveness, feasibility, scalability, sustainability, and integration within routine healthcare systems, particularly among rural populations and other underserved communities.

## Introduction and background

Diabetes mellitus has emerged as one of the fastest-growing non-communicable diseases worldwide and continues to pose substantial clinical, social, and economic challenges to health systems. Characterized by chronic hyperglycemia resulting from impaired insulin secretion, impaired insulin action, or both, diabetes affects multiple organ systems and is associated with long-term microvascular and macrovascular complications. The increasing prevalence of diabetes has been accompanied by a parallel rise in diabetes-related complications, creating an expanding burden on healthcare services, particularly in low- and middle-income countries (LMICs) where resources for prevention and long-term disease management remain limited [1,2].

Among the microvascular complications of diabetes, diabetic retinopathy represents one of the leading causes of preventable blindness and visual impairment among working-age adults. Persistent hyperglycemia progressively damages the retinal microvasculature, resulting in structural and functional changes that may ultimately culminate in irreversible vision loss if left undetected or untreated [3]. Although advances in retinal imaging and ophthalmic treatment have substantially improved visual outcomes, these benefits depend heavily on early diagnosis through routine retinal examination. Unfortunately, the asymptomatic nature of early-stage diabetic retinopathy means that many individuals remain unaware of retinal damage until visual impairment has already occurred [4].

Previous studies consistently indicate that awareness of diabetic retinopathy, knowledge of recommended eye examinations, and understanding of the consequences of delayed diagnosis are important determinants of screening uptake and preventive healthcare utilization [5]. Despite established screening recommendations, awareness remains inadequate in many settings, particularly among populations with limited access to health information and specialist eye care services.

These disparities are especially evident in rural communities, where geographic distance, shortages of trained eye-care professionals, financial limitations, transportation challenges, and lower health literacy collectively restrict access to preventive healthcare. In India, where a substantial proportion of the population resides in rural areas, these barriers contribute to delayed diagnosis and increased risk of preventable blindness among people living with diabetes [6]. Uttar Pradesh, India's most populous state, presents a particularly important setting for examining these challenges because of its large rural population, growing burden of diabetes, and persistent inequities in healthcare access.

Improving awareness has consequently become an important public health priority. Behavior change communication (BCC) recognizes that health outcomes are influenced not only by healthcare availability but also by individual knowledge, beliefs, perceived susceptibility, motivation, and social context [7]. Well-designed communication strategies have demonstrated their ability to strengthen health knowledge, influence attitudes, encourage preventive practices, and increase the utilization of screening services. As behavioral science has increasingly informed public health interventions, communication has evolved from simple information dissemination to theory-driven approaches that support sustained behavior change.

The rapid expansion of digital technology has created new opportunities for delivering health communication at population scale. Mobile health (mHealth), defined as the use of mobile and wireless technologies to support healthcare delivery and health promotion, has emerged as an accessible and cost-effective platform for behavior change interventions [8]. Mobile phones have become widely available across urban and rural settings, allowing health information to reach individuals directly regardless of geographical location. Messaging-based interventions, including text messages, multimedia communication, and application-based platforms, have demonstrated potential for improving health knowledge, reinforcing preventive behaviors, supporting treatment adherence, and facilitating greater engagement with healthcare services [9,10]. Their ability to deliver culturally appropriate information in local languages further enhances their relevance for underserved populations.

Although the effectiveness of mHealth interventions has been demonstrated across several chronic diseases, evidence specifically addressing diabetic retinopathy awareness remains comparatively limited. Much of the existing literature has focused on diabetes self-management, medication adherence, glycemic control, and lifestyle modification, while relatively fewer studies have examined the use of mobile-based communication to improve awareness of diabetic eye disease and promote retinal screening, particularly among rural populations in India. In addition, successful implementation of digital health programs depends not only on intervention effectiveness but also on adoption, implementation quality, user engagement, contextual adaptation, and long-term sustainability [11].

Against this background, the present narrative review critically synthesizes the available evidence on diabetic retinopathy awareness, screening behavior, barriers to preventive eye care, behavior change theories, mHealth interventions, and implementation frameworks relevant to diabetic retinopathy prevention. Particular emphasis is placed on the potential of mobile-based BCC interventions to improve awareness and promote early retinal screening among adults with diabetes in rural India. By integrating evidence across public health, ophthalmology, behavioral science, digital health, and implementation research, this review seeks to identify current knowledge, highlight persistent evidence gaps, and inform the design of future mobile-based interventions aimed at reducing avoidable vision loss among populations at greatest risk.

## Review

Methodology

This narrative review synthesizes current evidence on diabetes mellitus, diabetic retinopathy, awareness and knowledge of diabetic retinopathy, retinal screening practices, barriers to screening, BCC, and the role of mHealth interventions in improving diabetic retinopathy awareness and screening uptake. Particular emphasis was placed on studies from India and other LMICs, where the growing burden of diabetes and constraints in eye-care delivery make context-specific communication and implementation strategies especially relevant.

A comprehensive literature search was conducted using PubMed, Scopus, Web of Science, Embase, and Google Scholar. Search terms included combinations of "diabetes mellitus", "diabetic retinopathy", "diabetic retinopathy awareness", "diabetic retinopathy screening", "screening behaviour", "health communication", "behaviour change communication", "mobile health", "mHealth", "SMS", "WhatsApp", "digital health", "telemedicine", "health behaviour", "implementation science", "India", and "rural India". Boolean operators (AND/OR) were used to combine search terms, and reference lists of eligible publications were manually screened to identify additional relevant studies.

The primary search included articles published between January 2015 and March 2025. Earlier publications were retained when they represented landmark clinical guidelines, foundational behavioral theories, implementation frameworks, or seminal studies that remain central to understanding diabetic retinopathy screening, behavior change, and digital health interventions. This approach ensured that the review reflected recent evidence while preserving key conceptual and methodological contributions that continue to inform current research and practice.

Eligible publications included systematic reviews, meta-analyses, randomized and quasi-experimental studies, observational studies, qualitative research, implementation studies, clinical practice guidelines, consensus statements, and policy documents published in English. Editorials, conference abstracts without full publications, letters to the editor, opinion articles, and studies not directly related to diabetic retinopathy awareness, screening behavior, communication strategies, or digital health interventions were excluded.

Titles and abstracts were initially screened for relevance, followed by full-text review of potentially eligible articles. Where multiple publications addressed similar topics, preference was given to the most recent, comprehensive, or methodologically rigorous evidence. The final review synthesized 72 publications covering the epidemiology of diabetes and diabetic retinopathy, awareness and health literacy, screening behavior, behavioral determinants, communication approaches, mHealth interventions, digital health ecosystems, and implementation frameworks relevant to diabetic retinopathy prevention.

The evidence was synthesized using a thematic approach. Findings were organized into the following domains: (i) burden of diabetes mellitus and diabetic retinopathy; (ii) awareness, health literacy, and screening behavior; (iii) barriers to retinal screening; (iv) BCC strategies; (v) mHealth interventions for diabetic retinopathy awareness and screening; (vi) India's evolving digital health ecosystem; and (vii) implementation and technology adoption frameworks applicable to mHealth interventions. Rather than providing a study-by-study description, evidence was critically synthesized to identify consistent findings, areas of disagreement, implementation challenges, and research gaps that can inform future interventions and policy development.

Review

Diabetes Mellitus and Diabetic Retinopathy: An Expanding Public Health Challenge

Diabetes mellitus is one of the fastest-growing non-communicable diseases worldwide and represents a major challenge for health systems because of its lifelong management requirements and associated complications. It is characterized by chronic hyperglycemia resulting from impaired insulin secretion, impaired insulin action, or both, leading to progressive damage to multiple organ systems, including the eyes, kidneys, nerves, cardiovascular system, and blood vessels. Although diabetes comprises type 1, type 2, and gestational diabetes, type 2 diabetes accounts for approximately 90-95% of all diagnosed cases and continues to drive the global epidemic owing to its strong association with population aging, urbanization, obesity, unhealthy diets, and physical inactivity [12].

The burden of diabetes has increased substantially over recent decades, particularly in LMICs, where rapid demographic, nutritional, and lifestyle transitions have accelerated disease prevalence [13]. These changes are no longer confined to urban populations. Rural communities are experiencing similar epidemiological shifts, characterized by reduced physical activity, changing dietary habits, and increasing obesity, resulting in a narrowing of the traditional urban-rural gap in diabetes prevalence [14]. Current projections indicate that nearly 700 million people may be living with diabetes by 2045, with the greatest increase expected in resource-constrained settings where health systems are often inadequately prepared to provide long-term chronic disease management [15]. Beyond its clinical consequences, diabetes imposes substantial economic and social costs through lifelong treatment, productivity losses, disability, and management of chronic complications, emphasizing the importance of preventive care and early intervention [13,16].

India bears a disproportionate share of this burden and is home to one of the world's largest populations of individuals living with diabetes [17]. Once predominantly an urban condition, diabetes has become increasingly common in rural populations because of socioeconomic development, nutritional transition, and population aging [18]. This shift presents important challenges for healthcare delivery, as many rural areas continue to experience shortages of trained healthcare professionals, diagnostic services, and structured chronic disease management programs. Consequently, individuals frequently encounter delays in diagnosis, inconsistent follow-up, and high out-of-pocket expenditure, all of which contribute to poorer long-term outcomes [19]. These challenges are particularly relevant in Uttar Pradesh, where limited healthcare infrastructure, low health literacy, financial constraints, and restricted access to specialist services further compromise diabetes management [20]. At the same time, widespread mobile phone ownership provides an opportunity to strengthen health communication and support self-management through scalable digital interventions.

Among the long-term complications of diabetes, diabetic retinopathy remains a leading cause of preventable vision impairment and blindness among working-age adults [21]. Chronic hyperglycemia initiates progressive microvascular damage through endothelial dysfunction, capillary leakage, retinal ischemia, and vascular occlusion, eventually leading to retinal neovascularization and diabetic macular edema. Disease progression is influenced by several well-established factors, including duration of diabetes, poor glycemic control, hypertension, dyslipidemia, obesity, and smoking [22]. These observations highlight that prevention of vision loss requires comprehensive diabetes management that addresses both metabolic control and modifiable cardiovascular risk factors.

Clinically, diabetic retinopathy progresses from non-proliferative diabetic retinopathy, characterized by microaneurysms, retinal hemorrhages, and other microvascular abnormalities, to proliferative diabetic retinopathy, in which retinal ischemia stimulates pathological neovascularization and increases the risk of vitreous hemorrhage and tractional retinal detachment. Diabetic macular edema may occur at any stage and remains one of the principal causes of visual impairment among individuals with diabetes [16,23,24]. Although effective treatment is available, visual outcomes depend largely on timely detection before irreversible retinal damage has occurred.

The public health significance of diabetic retinopathy lies in its high prevalence and asymptomatic progression during the early stages. Approximately one-third of individuals with diabetes develop some degree of diabetic retinopathy, and a substantial proportion progress to vision-threatening disease if not detected and managed appropriately [16]. Because early retinal changes rarely affect vision, reliance on symptoms alone frequently results in delayed diagnosis. Regular retinal assessment, undertaken according to diabetes type, duration of disease, pregnancy status, retinopathy severity, and applicable clinical guidelines, remains central to preventing avoidable vision loss and preserving visual function [25].

Digital health has emerged as an important enabler of these strategies. The World Health Organization's Global Strategy on Digital Health advocates the responsible use of digital technologies to strengthen health systems, improve service delivery, and advance universal health coverage through equitable, secure, and evidence-based implementation [26]. Within diabetes care, digital communication platforms have the potential to improve health literacy, reinforce recommended screening practices, facilitate follow-up, and support continuity of care, particularly in underserved populations. However, their effectiveness depends on integration within existing health systems and on interventions that address the behavioral and contextual factors influencing healthcare utilization rather than technology alone.

Improving screening coverage, however, requires more than the availability of clinical services. Despite established recommendations and effective treatment options, participation in retinal screening remains suboptimal in many settings, particularly in LMICs, where awareness, access to specialist care, and continuity of follow-up are often limited [23,27,28]. Consequently, diabetic retinopathy should be considered not only an ophthalmic complication but also a public health challenge that requires coordinated strategies integrating diabetes management, patient education, systematic screening, and accessible eye-care services [23,29].

Collectively, the available evidence indicates that reducing diabetes-related vision loss requires a continuum of care encompassing diabetes prevention, optimal metabolic control, risk factor management, timely retinal assessment, and appropriate treatment. Increasing awareness and promoting sustained engagement with recommended eye-care services remain essential components of this continuum. In this context, mobile-based behavior change interventions offer a promising approach to strengthening communication and improving screening uptake, particularly in rural and resource-constrained settings where conventional health education programs have limited reach.

Awareness, Health Literacy, and Screening Behavior in Diabetic Retinopathy

Awareness and health literacy are closely related but distinct determinants of preventive health behavior. Awareness refers to an individual's knowledge of a disease and its risk factors, consequences, and preventive measures, whereas health literacy encompasses the ability to obtain, understand, evaluate, and apply health information to make appropriate healthcare decisions [30]. Together, these influence patients' engagement with healthcare services, adherence to recommended treatment, and participation in preventive care. Across chronic diseases, higher health literacy has consistently been associated with improved self-management, greater use of preventive services, and better clinical outcomes [29]. However, evidence suggests that awareness alone is insufficient to sustain preventive behavior, particularly when structural and behavioral barriers remain unaddressed.

This distinction is particularly relevant in diabetic retinopathy, where retinal damage often progresses without noticeable visual symptoms. Unlike acute illnesses that prompt immediate healthcare seeking, early diabetic retinopathy is largely asymptomatic, leading many individuals to underestimate their susceptibility and delay retinal examinations until vision deteriorates [31]. Consequently, effective patient education should extend beyond informing individuals that diabetes can affect the eyes to explaining why retinal assessment is recommended before symptoms develop.

Knowledge of diabetes-related eye complications is consistently associated with greater acceptance of retinal screening. Individuals who understand that persistent hyperglycemia can damage the retina and lead to irreversible vision loss are generally more likely to seek preventive eye care than those with limited knowledge [6]. Nevertheless, studies across diverse settings continue to report substantial gaps in awareness of diabetic retinopathy and its consequences, particularly in rural and underserved populations where access to health information and specialist eye-care services remains limited. These findings indicate that awareness of the disease itself remains inadequate despite the increasing prevalence of diabetes.

Evidence from India reflects a similar pattern. Although awareness of diabetes has improved, knowledge regarding diabetic retinopathy, the need for retinal assessment, and the consequences of delayed diagnosis remains limited, especially in rural communities [32]. Many individuals seek ophthalmic care only after experiencing visual impairment, by which time retinal damage may already be advanced. This delay reflects not only inadequate awareness but also limited opportunities for counselling during routine diabetes care, reinforcing the need to integrate eye health education into diabetes management services.

Awareness of screening recommendations represents another important determinant of preventive behavior. Individuals who understand the purpose and benefits of retinal examinations are generally more likely to participate in screening programs than those who perceive eye examinations as necessary only after visual symptoms develop [16]. However, recommendations for retinal assessment vary according to diabetes type, duration of disease, pregnancy status, existing retinopathy, and national or international clinical guidelines. Therefore, communication strategies should focus on helping patients understand when and why retinal examinations are appropriate for their individual circumstances rather than promoting a uniform screening message [16].

Despite the importance of awareness, knowledge alone does not consistently translate into screening behavior. Participation in retinal screening is influenced by a combination of individual, social, and health system factors, including accessibility of services, affordability, healthcare provider recommendations, previous healthcare experiences, perceived susceptibility, and competing personal priorities [17]. Consequently, many individuals who recognize the importance of glycemic control continue to overlook routine eye examinations, highlighting the gap between knowledge and sustained preventive practice.

Knowledge, attitude, and practice (KAP) studies provide further insight into this gap. Across different settings, these studies consistently demonstrate that although many individuals possess basic knowledge of diabetic retinopathy, this knowledge is often insufficient to motivate regular retinal examinations. Preventive behavior is shaped by a broader set of influences, including perceived risk of vision loss, cultural beliefs, family support, healthcare accessibility, and financial considerations. Positive attitudes towards retinal screening frequently emerge after individuals receive appropriate information, yet favorable attitudes alone rarely overcome practical barriers to accessing eye-care services.

Urban-rural disparities further illustrate the interaction between awareness and access. Urban populations generally report higher levels of awareness, greater knowledge of recommended retinal examinations, and higher screening uptake than rural populations, reflecting better access to healthcare facilities, specialist ophthalmologists, educational opportunities, and health promotion activities [33]. In contrast, rural communities often experience lower educational attainment, limited health literacy, fewer opportunities to receive accurate health information, and restricted access to specialist services [14]. These factors contribute to delayed diagnosis and lower utilization of preventive eye-care services despite the increasing burden of diabetes.

Collectively, the evidence indicates that improving awareness is a necessary but insufficient strategy for increasing retinal screening. Successful interventions must address the broader determinants of preventive behavior by combining health education with accessible screening services, effective provider communication, and systems that facilitate timely referral and follow-up. In this context, mHealth interventions offer a promising platform for delivering personalized and contextually relevant BCC at scale. By providing repeated, accessible, and culturally appropriate messages, digital communication can strengthen health literacy, reinforce the importance of retinal assessment, and support sustained engagement with preventive eye-care services, particularly in rural and resource-constrained settings where conventional health education programs have limited reach [34].

Barriers to Diabetic Retinopathy Screening and Management

Despite advances in the diagnosis and treatment of diabetic retinopathy, uptake of retinal screening and timely eye-care services remains suboptimal, particularly in LMICs. Evidence indicates that screening behavior is shaped by a complex interaction of socioeconomic, health system, behavioral, psychological, and cultural factors rather than by awareness alone. These barriers often coexist, creating cumulative disadvantages that delay diagnosis, reduce screening uptake, and increase the risk of preventable vision loss.

Socioeconomic constraints are among the most consistently reported barriers to retinal screening. Individuals with limited financial resources frequently prioritize immediate household needs over preventive healthcare, particularly because diabetic retinopathy remains asymptomatic in its early stages [12]. Although retinal screening is a cost-effective intervention, direct costs such as consultation fees and indirect costs including transportation, accommodation, and loss of wages discourage attendance, especially among rural and economically disadvantaged populations [35]. Lower educational attainment further compounds these challenges by limiting awareness of diabetic retinopathy, reducing understanding of screening recommendations, and restricting engagement with preventive eye-care services [14]. Consequently, socioeconomic disadvantage influences both access to care and the ability to utilize available services effectively.

Health system limitations further constrain screening uptake. Many LMICs face shortages of trained ophthalmologists, retinal specialists, diagnostic facilities, and organized screening programs, resulting in inadequate service coverage, particularly in rural and underserved areas [14]. Even where services exist, fragmented referral pathways, poor coordination between diabetes and eye-care services, prolonged waiting times, and inadequate follow-up reduce continuity of care. Studies from India consistently report lower access to retinal screening in rural areas, where specialist services remain concentrated in urban centers [32]. These findings suggest that expanding service availability alone is unlikely to improve screening unless referral systems and integration of eye care within routine diabetes management are also strengthened.

Communication between healthcare providers and patients is another important determinant of screening behavior. Individuals frequently rely on physicians and other healthcare professionals for information regarding diabetes-related complications and preventive care. When retinal screening is not routinely discussed during diabetes consultations, patients may underestimate their risk or remain unaware of recommended eye examinations [17]. Integrating counselling on diabetic eye disease into routine diabetes care, supported by effective referral mechanisms, is therefore essential for improving screening participation.

Behavioral and psychological factors also influence decisions to seek preventive eye care. Fear of vision loss, anxiety about ophthalmic examinations, denial of disease severity, and low perceived susceptibility discourage participation in screening programs, particularly among individuals without visual symptoms [36,37]. These observations are consistent with the Health Belief Model, which proposes that preventive behavior depends on perceived susceptibility, perceived severity, perceived benefits, and perceived barriers [38]. In the context of diabetic retinopathy, patients who underestimate their risk or perceive limited benefit from retinal examinations are less likely to participate in screening. Interventions that increase risk perception, reinforce the benefits of early detection, and reduce anxiety may therefore improve screening uptake.

Cultural beliefs and behavioral practices further shape healthcare utilization. In some communities, individuals delay seeking formal healthcare because of reliance on traditional remedies, competing family or occupational responsibilities, or the misconception that normal vision indicates healthy eyes [13,39]. Limited health literacy amplifies these challenges by reducing understanding of the progressive and asymptomatic nature of diabetic retinopathy, while poor adherence to diabetes self-management may also decrease engagement with preventive eye care [40]. These findings indicate that culturally appropriate health education should address prevalent misconceptions alongside factual knowledge.

Gender-related inequities add another dimension to screening behavior, particularly in rural settings. Women often face barriers related to financial dependence, domestic responsibilities, restricted mobility, and limited decision-making autonomy, whereas men may delay preventive care because of occupational commitments and lower engagement with health services [41]. These differences highlight the importance of gender-sensitive strategies that recognize the distinct challenges faced by both women and men.

Collectively, the evidence demonstrates that barriers to retinal screening are multidimensional and mutually reinforcing. Interventions that focus solely on improving awareness are unlikely to achieve sustained improvements unless accompanied by stronger health systems, accessible screening services, effective provider communication, and strategies that address behavioral, socioeconomic, and cultural determinants of healthcare utilization. Digital health interventions offer an opportunity to complement these efforts by providing culturally appropriate education, personalized reminders, and continued patient engagement. However, their effectiveness depends on integration with existing healthcare systems and on addressing the broader contextual factors that influence screening behavior. These considerations provide the foundation for designing theory-informed, context-specific mHealth interventions to improve diabetic retinopathy awareness and screening uptake.

BCC and Theoretical Foundations for Diabetic Retinopathy Prevention

Improving awareness of diabetic retinopathy and increasing participation in retinal screening require more than the provision of health information. Although knowledge is an essential prerequisite for preventive health behavior, evidence consistently shows that awareness alone rarely results in sustained behavior change. Individuals may understand the importance of retinal screening yet fail to seek preventive eye care because of perceived barriers, competing priorities, low risk perception, social influences, or limited confidence in accessing healthcare services. Consequently, BCC has emerged as a key public health strategy that combines behavioral science with targeted communication to influence knowledge, attitudes, motivation, and long-term health practices [42].

Unlike conventional health education, which primarily focuses on information dissemination, BCC adopts a more comprehensive approach by addressing the cognitive, emotional, social, and environmental factors that shape health-related decisions [43]. Within diabetic retinopathy prevention, BCC aims not only to improve knowledge of diabetes-related eye disease but also to increase perceived susceptibility to vision loss, reinforce the benefits of early detection, address misconceptions regarding retinal screening, and encourage sustained engagement with preventive eye-care services.

The design of effective BCC interventions is informed by established behavioral theories that explain why individuals adopt or avoid preventive health behaviors. Although these theories emphasize different determinants of behavior, they should be viewed as complementary rather than competing frameworks. Collectively, they provide a robust conceptual foundation for developing interventions that address the multiple factors influencing retinal screening behavior.

The Health Belief Model explains screening behavior through individuals' perceptions of susceptibility to disease, severity of its consequences, benefits of preventive action, and barriers to care [44]. In diabetic retinopathy, low perceived risk, the absence of visual symptoms, and misconceptions about the necessity of retinal examinations frequently reduce motivation to seek screening. The Health Belief Model therefore highlights the importance of communication strategies that increase risk perception, emphasize the asymptomatic progression of diabetic retinopathy, and reinforce the value of early detection.

The Theory of Planned Behavior extends this perspective by recognizing that healthcare decisions are also influenced by social norms and perceived behavioral control [45]. Family support, recommendations from healthcare providers, financial considerations, transportation, and service accessibility all affect an individual's intention to undergo retinal screening. This framework underscores the importance of involving families, strengthening provider-patient communication, and reducing practical barriers to care.

Similarly, Social Cognitive Theory emphasizes that behavior is shaped by the interaction between individual beliefs, environmental influences, and observational learning [46]. Patients are more likely to adopt preventive practices when they receive encouragement from healthcare providers, observe positive health behaviors within their community, and develop confidence in their ability to access services. Community engagement, peer support, and positive reinforcement therefore represent important components of behavior change interventions.

In contrast, the Transtheoretical Model recognizes that behavior change occurs progressively rather than as a single event [47]. Individuals differ in their readiness to participate in retinal screening, ranging from those who are unaware of diabetic retinopathy to those who require ongoing reinforcement to maintain regular eye examinations. Tailoring communication to an individual's stage of readiness through education, reminders, motivational messages, and follow-up support may therefore improve both initiation and maintenance of preventive behaviors. 

Although each framework emphasizes different behavioral determinants, their common message is that retinal screening behavior is influenced by far more than knowledge alone. Evidence consistently demonstrates that awareness interacts with perceived risk, self-efficacy, family support, healthcare accessibility, and environmental influences to determine whether individuals engage in preventive eye care. Consequently, interventions based on a single theoretical model may overlook important determinants of behavior, whereas integrating complementary constructs across behavioral theories provides a more comprehensive basis for intervention design.

These principles are particularly relevant to mHealth interventions. Digital communication platforms enable the delivery of personalized educational messages, reminders, motivational reinforcement, and cues to action that can simultaneously address multiple behavioral determinants. For example, repeated messages can improve knowledge and perceived susceptibility (Health Belief Model), reinforce positive social norms (Theory of Planned Behavior), strengthen self-efficacy through encouragement (Social Cognitive Theory), and provide continued support throughout different stages of behavior change (Transtheoretical Model). Integrating these theoretical constructs within mobile-based communication strategies has the potential to improve awareness, increase participation in retinal screening, and support sustained engagement with diabetes self-management.

Overall, behavioral science provides a strong theoretical foundation for designing diabetic retinopathy interventions that move beyond information dissemination towards sustained behavior change. Rather than viewing awareness as the sole objective, effective communication strategies should address the behavioral, social, and contextual factors that influence healthcare utilization. As digital health technologies become increasingly integrated into routine healthcare delivery, theory-informed mHealth interventions offer an opportunity to deliver scalable, contextually relevant, and patient-centered communication capable of improving diabetic retinopathy awareness and strengthening the uptake of retinal screening services.

mHealth and the Digital Health Ecosystem for Diabetic Retinopathy Prevention

The rapid expansion of digital technologies has transformed healthcare delivery by enabling continuous communication, remote service provision, and patient-centered management beyond conventional clinical settings. Among these technologies, mHealth has emerged as a scalable approach for health promotion, disease prevention, and chronic disease management. The widespread availability of mobile phones, improved telecommunications infrastructure, and declining communication costs have enabled health systems to deliver health information directly to individuals irrespective of geographical location, thereby reducing barriers associated with conventional healthcare delivery [10]. These characteristics are particularly relevant for chronic diseases such as diabetes mellitus, where sustained patient engagement and long-term behavioral reinforcement are essential for preventing complications.

The application of mHealth is especially relevant to diabetic retinopathy, a leading cause of preventable vision loss that frequently remains asymptomatic until advanced stages. Because early retinal damage rarely produces visual symptoms, effective prevention depends on maintaining regular communication with individuals living with diabetes to reinforce awareness, encourage timely retinal examinations, and promote adherence to recommended diabetes care. Conventional health education, which relies primarily on facility-based counselling or community outreach, often provides intermittent contact and may not adequately support long-term behavioral change. Mobile technologies address this limitation by enabling the repeated delivery of educational messages, appointment reminders, motivational prompts, and follow-up communication at relatively low cost [8]. Consequently, mHealth has become an increasingly important component of strategies aimed at improving diabetes management and retinal screening.

mHealth encompasses the use of mobile and wireless technologies, including text messaging, voice calls, smartphone applications, wearable devices, and teleconsultation platforms, to support health education, patient monitoring, disease prevention, and healthcare delivery. These technologies extend healthcare beyond episodic clinical encounters by facilitating continuous interaction between healthcare providers and patients. Depending on the intervention, mHealth can support medication adherence, appointment scheduling, self-monitoring, behavioral counselling, and follow-up while reducing dependence on physical healthcare infrastructure [10]. Importantly, the choice of communication modality should be guided by the needs of the target population. SMS remains one of the most widely implemented approaches because of its low cost and compatibility with basic mobile phones, whereas voice messages improve accessibility for populations with limited literacy. Smartphone applications and teleconsultation platforms provide additional functionality through multimedia education, personalized feedback, remote monitoring, and specialist consultation, although their use depends on digital access and user capability.

The rapid expansion of mHealth has been accompanied by growing evidence of its effectiveness in improving treatment adherence, health knowledge, appointment attendance, and self-management across a range of chronic diseases [8,10]. However, intervention effectiveness depends less on the technology itself than on how communication is designed and integrated into routine care. Programs that deliver concise, culturally appropriate, and behaviorally informed messages through repeated engagement are more likely to influence preventive practices than those relying solely on one-time information dissemination. These observations suggest that mHealth should be viewed primarily as a communication platform rather than a technological solution in isolation. The digital transformation accelerated during the COVID-19 pandemic, when teleconsultation, remote monitoring, and digital communication became integral components of healthcare delivery [11].

India provides a favorable environment for implementing mHealth interventions because of its rapid expansion in mobile phone ownership, digital connectivity, and internet access. Mobile communication has become deeply embedded in everyday life across both urban and rural populations, creating opportunities to deliver health promotion messages directly to communities that traditionally have limited access to specialist services [23]. Rather than a temporary response to the pandemic, these developments have established digital health as an important component of routine healthcare, particularly for chronic disease management. Digital platforms can complement existing healthcare systems by strengthening communication between providers and patients, supporting referral pathways, improving continuity of care, and facilitating community-based follow-up [48,49].

The expansion of mHealth has occurred alongside broader reforms within India's digital health ecosystem. National initiatives such as the Ayushman Bharat Digital Mission aim to establish interoperable digital health infrastructure through electronic health records, digital health identities, and secure information exchange across healthcare providers [50,51]. Complementing these reforms, the eSanjeevani telemedicine platform has expanded access to specialist consultations by enabling remote patient-provider and provider-provider interactions, thereby improving continuity of care and reducing geographical barriers to healthcare access [52]. At the state level, digital platforms such as eKavach support beneficiary tracking, digital reporting, and program monitoring by frontline health workers, strengthening service delivery and follow-up within public health programs [53,54]. Together, these initiatives provide an enabling environment for integrating mHealth into routine diabetes care and preventive eye health services.

An important component of this digital ecosystem is eKavach, a mobile application developed by the Government of Uttar Pradesh to strengthen healthcare delivery through digital monitoring and real-time reporting of pregnant women. Designed primarily for frontline health workers, the platform facilitates beneficiary tracking, digital record management, timely service delivery, and improved coordination across different levels of the healthcare system [55]. By reducing paperwork and enabling real-time data collection, eKavach enhances accountability and supports evidence-based decision-making in public health programs. These capabilities may be particularly relevant for diabetes management, in which regular follow-up and continuity of care remain essential for preventing long-term complications [56].

Government policies supporting digital health in Uttar Pradesh further reinforce these developments by emphasizing accessibility, affordability, quality of care, and preventive health services. Consistent with the objectives of the Ayushman Bharat Digital Mission, state initiatives promote electronic health records, telemedicine services, mHealth applications, and digital capacity building among frontline health workers [57].

These developments are particularly relevant for states such as Uttar Pradesh, where large rural populations, uneven healthcare infrastructure, and shortages of specialist ophthalmic services continue to limit access to preventive care. When integrated with frontline health workers and primary healthcare services, mHealth has the potential to reinforce retinal screening recommendations, deliver context-specific health education, and maintain engagement with individuals living with diabetes. However, successful implementation depends not only on the availability of digital infrastructure but also on user acceptance, organizational readiness, and integration within existing health systems. These considerations underscore the importance of technology adoption and implementation frameworks in the design and scale-up of mHealth interventions.

Technology Adoption and Implementation Frameworks for mHealth Interventions

The availability of digital technologies alone does not ensure successful implementation of mHealth interventions. Their effectiveness depends on user acceptance, organizational readiness, health system capacity, and the ability to integrate digital solutions into routine healthcare. Consequently, technology adoption models and implementation science frameworks provide complementary perspectives for understanding the factors that influence the uptake, sustainability, and scale-up of mHealth interventions [58].

The Technology Acceptance Model (TAM) and the Unified Theory of Acceptance and Use of Technology (UTAUT) are among the most widely used frameworks for explaining technology adoption in healthcare. TAM proposes that technology use is primarily influenced by two perceptions: usefulness and ease of use. Individuals are more likely to engage with digital health interventions when they believe the technology improves health outcomes without requiring substantial effort. UTAUT extends this concept by incorporating performance expectancy, effort expectancy, social influence, and facilitating conditions while recognizing that demographic characteristics, digital experience, and organizational support influence technology use [59-62]. Collectively, these models suggest that successful mHealth interventions require not only functional technology but also user-friendly interfaces, credible content, technical support, and integration within routine healthcare services.

While TAM and UTAUT focus on individual acceptance, the Diffusion of Innovation Theory explains how digital interventions spread within communities and health systems. Adoption depends on the perceived relative advantage of an innovation, its compatibility with users' needs and existing practices, simplicity, trialability, and observable benefits [63]. This perspective is particularly relevant for rural public health programs, where acceptance is often influenced by community norms, peer experiences, and trust in healthcare providers. Demonstrating the practical value of mobile communication through pilot implementation and community engagement may therefore facilitate wider adoption of digital health interventions.

Technology acceptance alone, however, does not guarantee successful implementation at scale. Frameworks such as the Reach, Effectiveness, Adoption, Implementation, and Maintenance (RE-AIM), Non-adoption, Abandonment, Scale-up, Spread, and Sustainability (NASSS), Consolidated Framework for Implementation Research (CFIR), and Practical, Robust Implementation and Sustainability Model (PRISM) extend evaluation beyond individual users by examining organizational, contextual, and system-level determinants of implementation [64-67]. RE-AIM emphasizes intervention reach, effectiveness, adoption, implementation fidelity, and long-term maintenance, providing a structured approach for evaluating public health impact. NASSS focuses on factors contributing to non-adoption, scale-up, and sustainability of digital technologies within complex healthcare systems, whereas CFIR and PRISM examine how intervention characteristics, organizational readiness, external environments, and implementation processes influence program success. Together, these frameworks highlight that digital interventions are most effective when adapted to local contexts, supported by healthcare organizations, and integrated within existing service delivery pathways.

Behavioral implementation is further strengthened through frameworks that explain how motivation translates into sustained health behavior. Self-Determination Theory emphasizes the roles of autonomy, competence, and social support in maintaining behavioral change, while the Behavior Change Wheel and Communication for Behavioral Impact (COM-B) model conceptualize behavior as the interaction between capability, opportunity, and motivation [68,69]. These frameworks are particularly relevant for mobile messaging interventions, which can improve knowledge, strengthen motivation, provide behavioral prompts, and reinforce self-management through repeated, personalized communication.

No single framework adequately captures the complexity of implementing mHealth interventions for diabetic retinopathy. Technology adoption models explain why individuals accept digital tools, behavioral theories explain how communication influences preventive practices, and implementation frameworks identify the organizational and contextual factors required for sustainability. Integrating these complementary perspectives provides a stronger conceptual foundation for designing, evaluating, and scaling mHealth interventions. Such an approach is particularly important for diabetic retinopathy prevention, where improvements in awareness and screening depend on effective communication, user engagement, health system integration, and sustained implementation within routine diabetes care.

Evidence on Mobile-Based Interventions for Diabetes and Diabetic Retinopathy

mHealth interventions have become an important component of chronic disease management by facilitating continuous patient engagement beyond conventional healthcare settings. For diabetes mellitus, where effective management requires sustained self-care, medication adherence, lifestyle modification, and regular follow-up, digital communication offers a practical approach to reinforce health behaviors and improve continuity of care [70]. Compared with conventional facility-based education, mHealth interventions enable repeated, personalized communication at relatively low cost, making them particularly suitable for resource-constrained settings with limited healthcare personnel.

Evidence accumulated over the past decade demonstrates that mHealth interventions improve several aspects of diabetes management, including medication adherence, self-monitoring, clinic attendance, glycemic control, and patient knowledge [70-72]. Among the available approaches, SMS remains the most extensively evaluated because of its simplicity, scalability, and compatibility with both basic and smart mobile phones. Voice messages, multimedia messaging, mobile applications, and teleconsultation platforms have further expanded opportunities for patient education, behavioral counselling, remote monitoring, and specialist consultation. Despite differences in delivery platforms, successful interventions consistently share common characteristics: they provide concise and understandable information, deliver repeated behavioral reinforcement, and are integrated within routine healthcare services rather than functioning as stand-alone communication tools.

The effectiveness of mobile messaging extends beyond information dissemination. Repeated exposure to behaviorally informed messages reinforces knowledge, addresses misconceptions, provides cues to action, and supports sustained engagement with recommended health practices. Interventions grounded in behavioral theory and adapted to local language, literacy, and cultural contexts have generally demonstrated greater acceptability and participant engagement than generic health education programs [70]. These findings highlight that communication strategy, message design, and contextual relevance are as important as the technology used to deliver the intervention.

Application of mHealth within eye care has expanded considerably through teleophthalmology, digital referral systems, appointment reminders, and patient education. Teleophthalmology has improved access to specialist consultations by supporting remote retinal assessment and strengthening referral pathways, particularly in geographically underserved areas [52]. Similarly, digital communication has been used to promote awareness of eye diseases, encourage routine eye examinations, and improve follow-up after referral. However, most published interventions have focused on improving service delivery and access to ophthalmic care rather than changing patient behavior related to diabetic retinopathy screening.

Evidence specific to diabetic retinopathy remains comparatively limited. Existing studies suggest that mobile reminders and educational messages can increase awareness of diabetes-related eye disease, improve attendance for retinal examinations, and strengthen adherence to follow-up recommendations. Nevertheless, these interventions are heterogeneous in their design, delivery platforms, outcome measures, and duration of follow-up, making direct comparison difficult [35,47,71]. Many studies have evaluated appointment reminders or telemedicine services rather than comprehensive behavior change interventions, while relatively few have examined whether improvements in knowledge translate into sustained screening behavior or long-term reductions in vision-threatening disease.

Several methodological limitations are evident across the literature. Most studies have been conducted in high-income settings or urban populations with relatively good digital access, limiting their generalizability to rural communities in LMICs. Follow-up periods are frequently short, behavioral outcomes are inconsistently measured, and implementation outcomes such as reach, adoption, fidelity, acceptability, cost, and sustainability are rarely evaluated. Furthermore, many interventions focus on diabetes self-management broadly, with diabetic retinopathy representing only one component of larger chronic disease programs [5,52,70-72]. Consequently, evidence remains insufficient to determine which communication strategies are most effective for improving retinal screening uptake across diverse populations and healthcare settings.

The available literature also indicates that technology alone is unlikely to improve preventive eye-care behavior. Interventions that combine digital communication with healthcare provider engagement, community participation, culturally appropriate messaging, and structured referral pathways generally report better outcomes than technology-based approaches implemented in isolation. These findings reinforce the importance of integrating mHealth within existing health systems and aligning digital communication with behavioral theory and implementation science rather than viewing technology as a stand-alone solution.

India offers a favorable environment for advancing this evidence base because of its expanding digital health infrastructure, widespread mobile phone penetration, and increasing integration of digital technologies within primary healthcare. However, evidence evaluating theory-informed, context-specific mobile behavior change interventions for diabetic retinopathy remains scarce, particularly in rural populations where awareness, access to specialist services, and screening uptake continue to be limited. Most available studies have focused on telemedicine or diabetes management rather than communication strategies designed specifically to improve diabetic retinopathy awareness and preventive screening.

Taken together, current evidence supports the potential of mHealth to strengthen diabetes care and improve preventive eye health. However, important knowledge gaps remain regarding the optimal design, implementation, and long-term effectiveness of behavior change interventions for diabetic retinopathy. Future research should move beyond demonstrating feasibility to evaluating interventions that integrate behavioral theory, culturally contextualized communication, and implementation science within routine public health systems. Such evidence is essential for developing scalable and sustainable strategies capable of improving retinal screening uptake and reducing avoidable vision loss among people living with diabetes, particularly in underserved rural settings.

Table 1 summarizes the original research studies relevant to diabetes, diabetic retinopathy awareness, screening practices, digital health interventions, and behavior change strategies. Secondary sources were not included in the evidence summary table but were used throughout the narrative to provide broader context, compare findings across studies, and support the interpretation of the available evidence.

**Table 1:** Summary of original research studies included in the narrative review

Study title	Author(s)	Country	Study design	Sample	Major findings	Relevance to present review
Diabetic patients' awareness of diabetic retinopathy symptoms and complications	Abdulaal et al. (2019) [4]	Saudi Arabia	Cross-sectional study	385	Most participants knew diabetes could affect vision, but awareness of diabetic retinopathy symptoms and screening remained inadequate	Demonstrates persistent knowledge gaps requiring awareness interventions
Diabetics retinopathy knowledge and awareness assessment among the type 2 diabetics	Almalki et al. (2018) [22]	Saudi Arabia	Cross-sectional study	253	Knowledge was associated with education, duration of diabetes, and glycemic control	Identifies determinants influencing diabetic retinopathy awareness
Knowledge, attitude, and practice regarding diabetic retinopathy screening and eye management among diabetics in Saudi Arabia	Alqahtani et al. (2023) [12]	Saudi Arabia	Cross-sectional study	1,391	Positive attitudes toward screening but variable knowledge and practice levels	Highlights behavioral factors influencing screening uptake
Knowledge, awareness, and eye care-seeking behavior in diabetic retinopathy: a cross-sectional study in Jeddah, Kingdom of Saudi Arabia	Fallatah (2018) [6]	Saudi Arabia	Cross-sectional study	380	Overall awareness was moderate, but knowledge of recommended screening intervals was poor	Demonstrates the gap between awareness and health-seeking behavior
Lack of awareness amongst community patients with diabetes and diabetic retinopathy: the Singapore Malay Eye Study	Huang et al. (2009) [7]	Singapore	Population-based cross-sectional study	712	Many participants were unaware of diabetic retinopathy despite having diabetes	Highlights the need for community awareness programs
Profile of sight-threatening diabetic retinopathy and its awareness among patients with diabetes mellitus attending a tertiary care center in Kashmir, India	Kaushik et al. (2021) [31]	India	Hospital-based cross-sectional study	625	Poor awareness and delayed presentation were associated with sight-threatening disease	Reinforces the importance of early education and screening
Knowledge, attitude and practices on diabetes, hypertension and diabetic retinopathy and the factors that motivate screening for diabetes and diabetic retinopathy in a pyramidal model of eye health care	Lingam et al. (2018) [13]	India	Cross-sectional survey	202	Awareness was higher among diabetics, but adherence to regular retinal examination remained low	Demonstrates barriers between knowledge and practice
Knowledge of diabetes and diabetic retinopathy among rural populations in India, and the influence of knowledge of diabetic retinopathy on attitude and practice	Rani et al. (2008) [14]	India	Community-based cross-sectional study	1,260	Better knowledge significantly improved attitudes and screening practices	Provides evidence linking awareness to screening behavior
Prevalence, awareness, treatment and control of diabetes in India from the countrywide national NCD monitoring survey	Mathur et al. (2022) [19]	India	National cross-sectional survey	12,000 households	Diabetes awareness and disease control remain suboptimal nationwide	Justifies large-scale awareness interventions
Perception of care and barriers to treatment in individuals with diabetic retinopathy in India: 11-city 9-state study	Shukla et al. (2016) [3]	India	Multi-center cross-sectional study	3,579	Cost, poor awareness, and accessibility were major barriers to treatment	Identifies health system barriers affecting diabetic retinopathy care
Awareness of diabetic retinopathy among diabetes mellitus patients visiting a hospital of North India	Singh et al. (2022) [42]	India	Hospital-based cross-sectional study	272	Overall awareness of diabetic retinopathy remained low despite regular diabetes care	Supports integrating diabetic retinopathy education into routine diabetes services
Prevalence of diabetic retinopahty in India: results from the National Survey 2015-19	Vashist et al. (2021) [24]	India	National population-based survey	57,808	Provided national estimates of diabetic retinopathy prevalence	Demonstrates the public health burden requiring improved screening
Does a diabetic retinopathy educational program raise awareness among elderly diabetic patients?	Khalaf et al. (2019) [30]	Egypt	Quasi-experimental pre-post study	200	Knowledge and attitudes improved significantly following structured education	Supports patient education programs for behavior change
Effectiveness of health education and monetary incentive on uptake of diabetic retinopathy screening at a community health center in South Gujarat, India	Chariwala et al. (2020) [17]	India	Community intervention study	462	Combined education and incentives significantly increased screening attendance	Demonstrates effective community-level intervention strategies
Diabetic retinopathy screening model for rural population: awareness and screening methodology	Rani et al. (2005) [18]	India	Community implementation study	Rural program	Community-based screening model successfully increased awareness and uptake	Demonstrates feasibility of rural screening programs
A mobile phone informational reminder to improve eye care adherence among diabetic patients in rural China: a randomized controlled trial	Chen et al. (2018) [35]	China	Randomized controlled trial	233	SMS reminders significantly improved attendance for diabetic eye examinations	Provides strong evidence supporting mobile messaging interventions
Preliminary effectiveness and feasibility of ASHA-led mobile health intervention for diabetes care in Indian primary health care settings	Bassi et al. (2025) [72]	India	Cluster randomized controlled trial	100	Improved HbA1c, medication adherence, and healthcare utilization	Demonstrates effectiveness of community health worker-led mHealth interventions
Factors influencing patient adherence with diabetic eye screening in rural communities: a qualitative study	Liu et al. (2018) [36]	USA	Qualitative study	29	Transportation, communication, and provider recommendations influenced adherence	Identifies behavioral and contextual barriers to screening
Impact of diabetic retinopathy awareness training on community health workers' knowledge and referral practices in Fiji: a qualitative study	Ram et al. (2022) [40]	Fiji	Qualitative study	Community health workers	Training improved knowledge, confidence, and referral practices	Demonstrates the value of frontline health worker capacity building
Prevalence of diabetic retinopathy in India stratified by known and undiagnosed diabetes, urban-rural locations, and socioeconomic indices: results from the SMART India population-based cross-sectional screening study	Raman et al. (2022) [41]	India	Population-based cross-sectional study	42,146	Reported prevalence across urban-rural and socioeconomic groups	Provides robust epidemiological evidence supporting systematic screening
Estimating the need for diabetic retinopathy services in north India: evidence from a population-based survey in the catchment population of an eye care provider in central Uttar Pradesh	Sabherwal et al. (2025) [20]	India	Population-based survey	20,955	Quantified service needs and highlighted unmet screening demand	Supports planning of diabetic retinopathy screening services
Public health system integration of avoidable blindness screening and management, India	Gudlavalleti et al. (2018) [29]	India	Health systems implementation study	Program evaluation	Demonstrated feasibility of integrating eye screening into primary healthcare	Supports integration of diabetic retinopathy screening within health systems
Impact of mobile health in diabetic retinopathy awareness and eye care behavior among Indigenous women	Umaefulam and Premkumar (2020) [47]	Canada	Intervention study	Indigenous women	mHealth improved awareness and eye care practices	Supports mHealth approaches among underserved populations
Prospects of mobile technology among community health workers and health beneficiaries in rural India	Dubey and Tripathi (2025) [53]	India	Cross-sectional study	Rural participants	High acceptance and perceived usefulness of mobile technologies	Supports scalability of mobile-based behavior change interventions
Understanding factors influencing elderly diabetic patients' continuance intention to use digital health wearables: extending the Technology Acceptance Model (TAM)	Ahmad et al. (2020) [59]	Pakistan	Cross-sectional survey	320	Perceived usefulness and ease of use significantly predicted continued technology adoption	Provides theoretical support using the Technology Acceptance Model (TAM)
Factors influencing the acceptance of telemedicine for diabetes management	Rho et al. (2015) [61]	South Korea	Cross-sectional survey	216	Social influence, trust, and perceived usefulness significantly predicted telemedicine acceptance	Supports behavioral determinants relevant to digital health adoption

Research Gaps and Future Directions

Despite substantial progress in the understanding, diagnosis, and treatment of diabetic retinopathy, important evidence and implementation gaps continue to limit the effectiveness of prevention programs, particularly in LMICs. Although the literature consistently identifies inadequate awareness, delayed screening, and poor utilization of eye-care services as major contributors to avoidable vision loss, comparatively less evidence is available on interventions capable of producing sustained improvements in screening behavior and long-term preventive care.

A major limitation of the current evidence base is its emphasis on describing awareness deficits rather than evaluating strategies to address them. Numerous studies have documented poor knowledge of diabetic retinopathy and low uptake of retinal screening among people living with diabetes, yet most have employed cross-sectional designs that provide limited insight into how awareness can be translated into sustained behavioral change. Evidence reviewed in this manuscript indicates that knowledge alone is insufficient to improve screening attendance because healthcare decisions are influenced by a combination of behavioral, socioeconomic, cultural, and health system factors. However, relatively few interventions have systematically incorporated behavioral theories to address these multidimensional determinants. Greater application of frameworks such as the Health Belief Model, Theory of Planned Behavior, Social Cognitive Theory, and Transtheoretical Model could strengthen intervention design by targeting risk perception, self-efficacy, social influences, and readiness for behavior change rather than relying solely on information dissemination.

A second gap relates to the limited focus on diabetic retinopathy within the broader digital health literature. While mHealth interventions have demonstrated benefits for diabetes self-management, medication adherence, lifestyle modification, and glycemic control, evidence specific to diabetic retinopathy awareness and retinal screening remains comparatively sparse. Existing studies frequently assess general diabetes outcomes, with limited evaluation of whether digital communication improves retinal screening uptake, follow-up adherence, or early detection of sight-threatening disease. Given that diabetic retinopathy prevention depends on lifelong surveillance, even in asymptomatic individuals, there is a need for interventions specifically designed to promote screening behavior and sustained engagement with preventive eye care.

The geographical concentration of available evidence presents an additional challenge. Much of the published literature originates from high-income countries or urban healthcare settings with greater access to specialist services, digital infrastructure, and organized screening programs. Consequently, the transferability of these findings to rural populations in LMICs remains uncertain. In India, although mobile phone penetration and digital connectivity have expanded rapidly, rural communities continue to experience barriers related to health literacy, specialist access, transportation, and socioeconomic disadvantage. Future research should therefore prioritize context-specific interventions that account for local healthcare systems, community beliefs, literacy levels, and patterns of technology use.

The growing digital health ecosystem in India offers opportunities that remain largely underexplored in diabetic retinopathy prevention. National initiatives such as the Ayushman Bharat Digital Mission, eSanjeevani, and state-level digital health platforms have created infrastructure capable of supporting longitudinal patient engagement, referral tracking, and integrated care. However, there is limited evidence on how these platforms can be leveraged to improve diabetic retinopathy awareness, strengthen referral pathways, or support routine retinal screening within existing public health programs. Future studies should move beyond stand-alone digital interventions and examine how mobile communication can be integrated with primary healthcare services, non-communicable disease programs, telemedicine platforms, and community-based health workers.

Another important gap concerns implementation and scale-up. Most studies focus on intervention effectiveness, while comparatively little attention is paid to adoption, implementation fidelity, sustainability, cost-effectiveness, and integration within routine healthcare systems. As digital health programs transition from pilot projects to population-level interventions, implementation outcomes become increasingly important. Frameworks such as RE-AIM, CFIR, PRISM, and NASSS offer valuable approaches for evaluating intervention reach, organizational readiness, scalability, and long-term sustainability, yet their application within diabetic retinopathy programs remains limited. Incorporating implementation science into future research will help identify the contextual and organizational factors that determine whether effective interventions can be successfully scaled and sustained.

The literature also highlights the importance of culturally responsive communication. Evidence suggests that digital interventions are more effective when messages are concise, behaviorally informed, culturally relevant, and delivered in local languages. Nevertheless, few studies have systematically evaluated culturally adapted BCC strategies for diabetic retinopathy in rural Indian settings. Future interventions should incorporate local social contexts, community norms, family influences, and frontline health workers such as accredited social health activists (ASHAs) to enhance acceptability, engagement, and program reach.

Finally, methodological limitations continue to constrain the evidence base. Considerable heterogeneity exists in intervention design, outcome measures, follow-up duration, and evaluation methods, limiting comparison across studies and synthesis of findings. Many interventions report short-term improvements in awareness or knowledge but provide limited evidence regarding sustained behavioral change, long-term screening adherence, clinical outcomes, or program sustainability. Future research should prioritize robust study designs, standardized outcome measures, longer follow-up periods, and evaluation of both behavioral and implementation outcomes.

Addressing these gaps will require multidisciplinary approaches that integrate behavioral science, digital health, implementation science, and public health practice. Theory-informed, culturally contextualized mHealth interventions embedded within existing healthcare systems offer considerable potential to improve diabetic retinopathy awareness, strengthen retinal screening uptake, and reduce avoidable vision loss. Future research should focus not only on intervention effectiveness but also on scalability, sustainability, integration, and real-world implementation within routine healthcare delivery, particularly in underserved populations where the burden of preventable visual impairment remains greatest.

## Conclusions

Diabetic retinopathy continues to represent one of the leading causes of preventable visual impairment among individuals living with diabetes despite the availability of effective screening programs and evidence-based treatment. The literature reviewed in this manuscript demonstrates that delayed diagnosis remains closely associated with inadequate awareness, limited health literacy, behavioral barriers, socioeconomic inequalities, and challenges within healthcare systems. These factors interact to reduce participation in retinal screening and delay timely treatment, particularly among rural and underserved populations.

The review further indicates that improving awareness alone is insufficient to achieve sustained behavioral change. Preventive eye-care practices are influenced by a complex interaction of cognitive, behavioral, social, cultural, and structural determinants that require comprehensive intervention strategies. Behavior change theories provide important conceptual guidance for understanding these determinants and designing communication programs that extend beyond information dissemination towards sustained engagement with preventive healthcare.

The emergence of mHealth has created unprecedented opportunities to strengthen diabetic retinopathy prevention through continuous, personalized, and scalable communication. Increasing mobile phone penetration, expanding digital health infrastructure, and supportive national initiatives such as the Ayushman Bharat Digital Mission provide a favorable environment for integrating mobile-based health communication into routine diabetes care. Mobile messaging, telemedicine, and digital health platforms have demonstrated considerable promise in improving health education, supporting self-management, reinforcing preventive behaviors, and expanding access to healthcare services.

However, important evidence gaps remain. Few interventions have specifically focused on diabetic retinopathy awareness using theory-informed mobile behavior change strategies, particularly within rural populations of India. Furthermore, limited evidence is available regarding long-term implementation, scalability, sustainability, and integration within existing public health systems. These gaps highlight the need for context-specific interventions that combine behavioral science with digital health while accounting for local healthcare infrastructure, cultural characteristics, and socioeconomic realities.

Overall, the available evidence suggests that mobile-based behavior change interventions represent a promising strategy for improving awareness of diabetic retinopathy, increasing retinal screening uptake, and strengthening preventive eye-care practices among individuals living with diabetes. Future research should focus on developing and rigorously evaluating culturally appropriate, theory-driven, and implementation-oriented digital health interventions capable of reducing avoidable vision loss while supporting equitable access to preventive eye care in resource-constrained settings.
